# Low-Concentration Tributyltin Decreases *GluR2* Expression via Nuclear Respiratory Factor-1 Inhibition

**DOI:** 10.3390/ijms18081754

**Published:** 2017-08-11

**Authors:** Keishi Ishida, Kaori Aoki, Tomoko Takishita, Masatsugu Miyara, Shuichiro Sakamoto, Seigo Sanoh, Tomoki Kimura, Yasunari Kanda, Shigeru Ohta, Yaichiro Kotake

**Affiliations:** 1 Graduate School of Biomedical and Health Sciences, Hiroshima University, 1-2-3 Kasumi, Minami-ku, Hiroshima 734-8553, Japan; d146668@hiroshima-u.ac.jp (K.I.); p5r1an2wh3y.4.bdbe@gmail.com (K.A.); takishita-tomoko@pmda.go.jp (T.T.); miyara128@hiroshima-u.ac.jp (M.M.); shuichiro.sakamoto@abbvie.com (S.S.); sanoh@hiroshima-u.ac.jp (S.S.); sohta@hiroshima-u.ac.jp (S.O.); 2 Research Fellow of the Japan Society for the Promotion of Science, 5-3-1 Kojimachi, Chiyoda-ku, Tokyo 102-0083, Japan; 3 Faculty of Science and Engineering, Setsunan University, 17-8 Ikedanakamachi, Neyagawa 572-8508, Japan; tomoki@lif.setsunan.ac.jp; 4 Division of Pharmacology, National Institute of Health Sciences, 1-18-1, Kamiyoga, Setagaya-ku, Tokyo 158-8501, Japan; kanda@nihs.go.jp

**Keywords:** tributyltin, *GluR2*, nuclear respiratory factor-1, neuronal vulnerability

## Abstract

Tributyltin (TBT), which has been widely used as an antifouling agent in paints, is a common environmental pollutant. Although the toxicity of high-dose TBT has been extensively reported, the effects of low concentrations of TBT are relatively less well studied. We have previously reported that low-concentration TBT decreases α-amino-3-hydroxy-5-methylisoxazole-4-propionic acid (AMPA)-type glutamate receptor subunit 2 (*GluR2*) expression in cortical neurons and enhances neuronal vulnerability to glutamate. However, the mechanism of this TBT-induced *GluR2* decrease remains unknown. Therefore, we examined the effects of TBT on the activity of transcription factors that control *GluR2* expression. Exposure of primary cortical neurons to 20 nM TBT for 3 h to 9 days resulted in a decrease in *GluR2* mRNA expression. Moreover, TBT inhibited the DNA binding activity of nuclear respiratory factor-1 (NRF-1), a transcription factor that positively regulates the *GluR2*. This result indicates that TBT inhibits the activity of NRF-1 and subsequently decreases *GluR2* expression. In addition, 20 nM TBT decreased the expression of genes such as cytochrome c, cytochrome c oxidase (COX) 4, and COX 6c, which are downstream of NRF-1. Our results suggest that NRF-1 inhibition is an important molecular action of the neurotoxicity induced by low-concentration TBT.

## 1. Introduction

Organotin compounds, especially tributyltin chloride (TBT), have been widely used as biocides, agricultural fungicides, and antifouling agents in paints. Because of its widespread use as an antifouling agent on the hulls of ships, toxic levels of TBT have been released into water and are polluting marine environments. Although the use of TBT in paints is currently internationally regulated, the issue of secondary exposure from contaminated fish, solids, and water remains, owing to its high bioaccumulation and long-term environmental persistence [1,2]. Indeed, about 200–600 ng/g TBT has been detected in fish serum [3]; TBT has also been detected in human blood [4]. Whalen et al. have reported TBT concentrations between 10 and 100 nM in human blood [5]. Since the 1980s, the toxicity of TBT, such as its disruption of the endocrine system, has been reported. It is well known that TBT causes irreversible alteration of the reproductive organs in some female gastropods, known as imposex [6], and some studies have proposed that TBT inhibits aromatase enzymes, which are responsible for the conversion of androgen to estrogen [7,8]. Although the toxic effects of TBT have been well studied, most of these effects have been observed at micromolar levels of TBT, which generally result in cell death within 24 h in mammalian cells. The mechanisms of TBT-induced toxicity at high concentrations may therefore be different from those at low concentrations that do not induce cell death. Thus, it is important to clarify the specific mechanisms of toxicity induced by low concentrations of TBT.

Some studies on the underlying mechanisms of TBT-induced toxicity without cell death have already been reported. For example, TBT is known to be a potential agonist of peroxisome proliferator-activated receptor (PPAR) γ and retinoid X receptor (RXR), and activates these nuclear receptors without inducing apoptosis or necrosis at 10–100 nM in mammalian cells [9]. Nakanishi suggested that RXR activation is an important mechanism in TBT-induced endocrine disruption in gastropods, because 9-*cis* retinoic acid (a well-known RXR ligand) induces gastropod imposex [10,11].

Neurotoxic effects of organotin compounds, including TBT, have also been reported in several studies. For example, a study in which TBT was orally administered to rats showed that TBT can pass through the blood-brain barrier (BBB) [12]. This means that, following intake through contaminated seafood, TBT can be delivered to the brain and adversely affect the central nervous system (CNS). TBT compounds have also been shown to alter the levels of neurotransmitters in the midbrain of mice [13], and induce behavioral abnormality [14]. Konno et al. demonstrated that TBT can affect *N*-methyl-d-aspartate (NMDA)-type glutamate receptors in preweanling mice [15]. In addition, we have previously reported the toxicity of nanomolar TBT to mammalian cells [16,17,18]. Exposure of rat cortical neurons to a low level (20 nM) of TBT decreased the expression of α-amino-3-hydroxy-5-methylisoxazole-4-propionic acid (AMPA)-type glutamate receptor subunit 2 (*GluR2*), and increased neuronal vulnerability to glutamate without inducing neuronal death [16]. The *GluR2* decrease was also observed in mammalian brain by prenatal exposure to TBT [19]. The Ca^2+^ permeability of the AMPA receptor is dependent upon the *GluR2* subunit, and *GluR2* plays a role in blocking Ca^2+^ influx into the cytoplasm at a steady state. Neuronal cells that contain AMPA receptors lacking the *GluR2* subunit show high Ca^2+^ permeability and vulnerability to excitotoxicity [20]. Thus, the effect of TBT on *GluR2* may have an important role in the underlying mechanism of TBT-induced toxicity at low concentrations.

In support of this, we have recently reported that a decrease in *GluR2* expression, and a subsequent enhancement in neuronal vulnerability, is similarly induced by some other environmental pollutants [21,22,23,24]. Perfluorooctane sulfonate (PFOS), a surfactant, decreases *GluR2* expression and enhances neuronal vulnerability to excitotoxicity both in vivo and in vitro [25]. *GluR2* expression in the rat cerebral cortex was decreased following developmental exposure to PFOS, and neuronal death was subsequently induced by injecting kainate (a glutamate analog) into the PFOS-exposed rats [25]. These findings suggest that a decrease of *GluR2* in the brain is involved in neurotoxicity induced by environmental chemicals. However, the mechanism via which environmental chemicals like TBT induce a decrease in *GluR2* remains unknown. Therefore, in the present study, we focused on the effect of TBT on transcription factors to clarify the mechanism of TBT-induced *GluR2* decreases. We here demonstrate that TBT-induced decreases in *GluR2* are mediated by decreased transcriptional activity of nuclear respiratory factor-1 (NRF-1), a transcription factor that positively regulates the *GluR2*.

## 2. Results

### 2.1. Effect of TBT Exposure on GluR2 Expression

**Figure 1:**
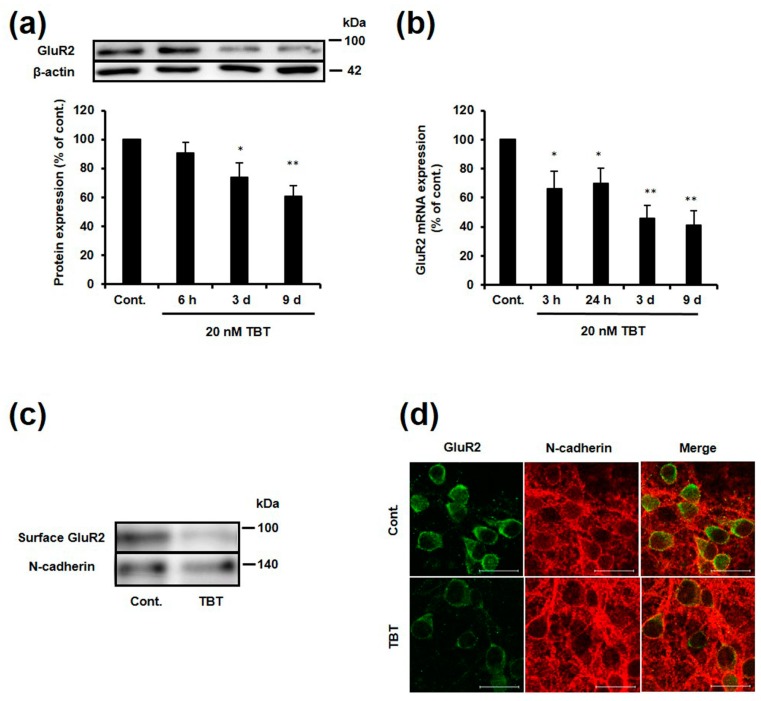
TBT-induced changes in *GluR2* whole cell protein, mRNA, and membrane protein expression. (**a**) Cortical neurons were exposed to DMSO (as a control) or 20 nM TBT for various times, and then *GluR2* protein expression was detected by western blotting. Quantitative analysis was performed using Image J software, and the *GluR2* protein levels were corrected based on the β-actin protein levels. The data are expressed as the mean + S.E.M. (*n* = 5). * *p* < 0.05 vs. control, ** *p* < 0.01 vs. control; (**b**) Cortical neurons were exposed to DMSO or 20 nM TBT for various times, and then *GluR2* mRNA expression was measured by real-time PCR. The mRNA level was normalized to glyceraldehyde-3-phosphate dehydrogenase (GAPDH). The data are expressed as the mean + S.E.M. (*n* = 3), * *p* < 0.05 vs. control, ** *p* < 0.01 vs. control; (**c**) Cortical neurons were exposed to DMSO or 20 nM TBT for 9 days. The cell surface proteins were then biotinylated, and detected by western blotting; (**d**) Cortical neurons were exposed to DMSO or 20 nM TBT for 9 days, and immunostaining was performed using a mouse anti-*GluR2* antibody that recognizes the *N*-terminal extracellular domain of *GluR2* (green), and a rabbit anti-*N*-cadherin antibody (red). Yellow indicates co-localization of *GluR2* and *N*-cadherin. Scale bar: 20 μm.

We have previously reported that *GluR2* protein and mRNA expression is decreased in rat primary cortical neurons following exposure to 20 nM TBT for 9 days [16]. However, it is currently unknown how long it takes for *GluR2* expression to be decreased following TBT exposure. To investigate the time-dependent effect of TBT exposure on *GluR2* expression, rat cortical neurons were exposed to 20 nM TBT for various lengths of time, and then protein and mRNA expression was measured. As shown in Figure 1a, the protein expression of *GluR2* significantly decreased to 74% and 60%, respectively, relative to the control, following exposure to 20 nM TBT for 3 and 9 days. The *GluR2* mRNA expression significantly decreased to 66%, 69%, 46%, and 42%, respectively, relative to the control after 3 h, 24 h, 3 days, and 9 days of TBT exposure (Figure 1b). Therefore, a time-dependent decrease of both *GluR2* protein and mRNA expression was observed in the neurons exposed to TBT. *GluR2* functions as a component of AMPA receptors in the plasma membrane; therefore, we next investigated the *GluR2* expression in the membrane by western blotting. The *GluR2* expression in the plasma membrane was decreased by TBT exposure (Figure 1c). This was supported by the results of an immunostaining experiment, in which expression of *GluR2* was visualized alongside the membrane protein marker *N*-cadherin (Figure 1d).

Next, we investigated whether TBT enhances neuronal vulnerability to glutamate. After TBT exposure, cortical neurons were exposed to 50–100 μM glutamate for 24 h and cell viability was measured. Cell viability of TBT-exposed neurons was significantly decreased by 50 μM glutamate treatment compared to that of control neurons. Moreover, this enhanced susceptibility to glutamate was attenuated by 1-naphthyl acetyl spermine (NAS, a specific Ca^2+^-permeable (*GluR2* lacking) AMPA receptor blocker) (Figure S1). These results suggest that the decrease in *GluR2* protein expression induced by TBT is attributable to transcriptional repression of *GluR2* mRNA, which leads to decreased membrane *GluR2* expression and increased neuronal vulnerability.

### 2.2. Effect of TBT Exposure on GluR2 Expression

**Figure 2:**
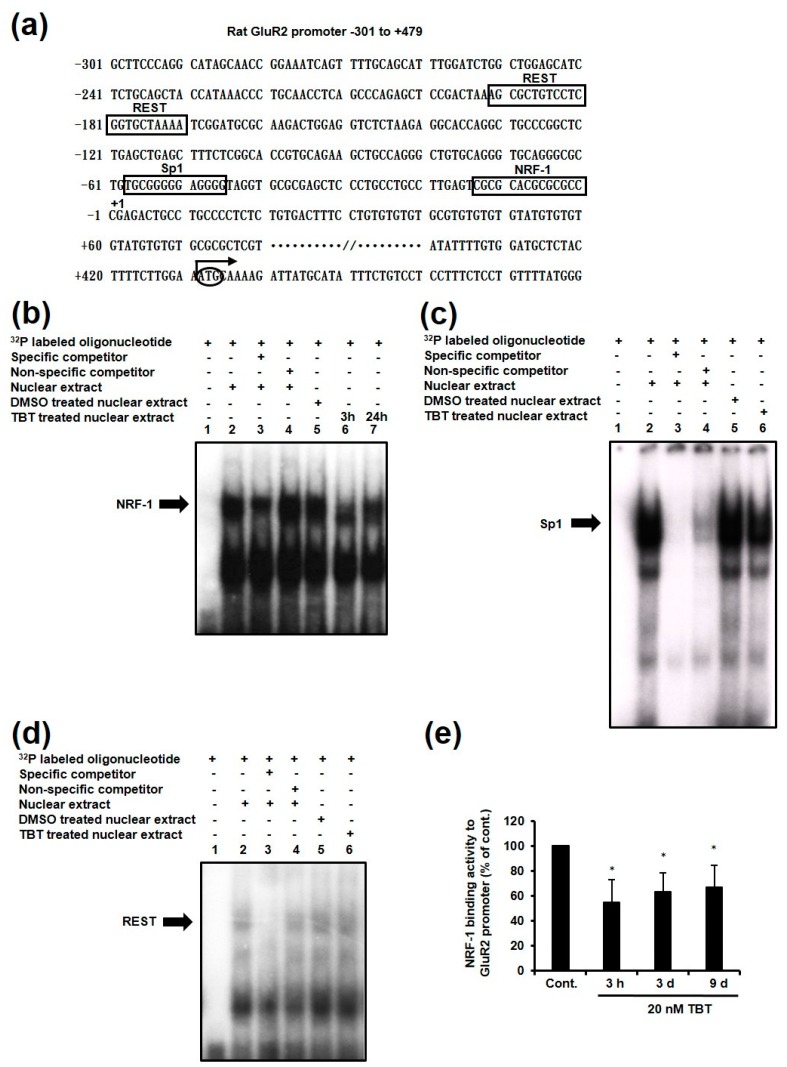
Effect of TBT on the binding activity of NRF-1, Sp1, and REST to their putative binding sites. (**a**) Partial nucleotide sequences of the rat *GluR2* region, and the binding sites of NRF-1, Sp1, and REST. The 5′-most prominent initiation site is shown as the +1 transcription initiation site, located 430 bases 5′ of the *GluR2* AUG (bent arrow). Nuclear proteins were extracted from DMSO- or TBT-exposed cortical neurons, and the DNA binding activities of NRF-1 (**b**), Sp1 (**c**), and REST (**d**) were analyzed by EMSA. An excess of specific (lane 3) or non-specific (lane 4) oligonucleotides was added as a competitor. DMSO-treated nuclear extracts (lane 5) and TBT-treated nuclear extracts (lane 6 and 7) were incubated with 32P-labelled oligonucleotides containing the *GluR2* promoter region, and DNA–protein complexes were separated from free DNA probes by gel electrophoresis. The arrows show specific band shifts; (**e**) Cortical neurons were exposed to DMSO as a control or 20 nM TBT for various times, and then the NRF-1 binding activity to the *GluR2* promoter was examined by ChIP assay using NRF-1 antibody. Purified DNA samples were subjected to quantitative real-time PCR analyses, and the DNA levels were corrected relative to the input sample. The data are expressed as the mean + S.E.M. (*n* = 5). * *p* < 0.05 vs. control.

It is known that *GluR2* mRNA expression is regulated by a variety of transcription factors, including NRF-1, Sp1 and RE-1 silencing transcription factor (REST), also known as neuron restrictive silencer factor (NRSF) [26] (Figure 2a). To clarify the mechanism of the TBT-induced *GluR2* decrease, we examined the binding activity of these three transcription factors to their putative binding sites on the *GluR2* promoter using an electrophoresis mobility shift assay (EMSA). An oligonucleotide that included the NRF-1 binding sequence was incubated with nuclear extracts collected from TBT-treated cells, or dimethyl sulfoxide (DMSO)-treated cells as a control (Figure 2b). Following TBT exposure for 3 h (Figure 2b, lane 6) and 24 h (Figure 2b, lane 7), band shifts were observed compared with the control (Figure 2b, lane 5). In contrast, the band shifts for Sp1 (Figure 2c, lane 6) and REST (Figure 2d, lane 6) were not changed by TBT exposure (Figure 2c, lane 5 and Figure 2d, lane 5). These results suggest that only the binding activity of NRF-1 to its putative binding site was decreased, and that the binding activities of Sp1 and REST were not changed by TBT exposure. Furthermore, we performed a chromatin immunoprecipitation (ChIP) assay to determine whether TBT influences the binding of NRF-1 to the *GluR2* promoter in rat primary cortical neurons. Real time PCR measurements indicated that the number of NRF-1 antibody-immunoprecipitated DNA fragments containing the *GluR2* promoter was decreased to about 60% relative to the control following TBT exposure for 3 h, 3 days, and 9 days (Figure 2e), which was consistent with the EMSA data shown in Figure 2b. These results suggest that TBT specifically inhibits the transcription factor activity of NRF-1, without affecting the activity of Sp1 and REST.

### 2.3. Effect of TBT on the Expression of NRF-1 Downstream Genes

**Figure 3:**
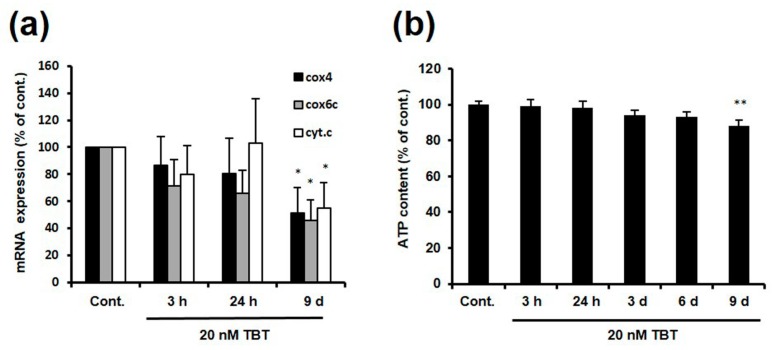
TBT-induced changes in COX4, COX6c, and cyt.c mRNA expression, and intracellular ATP content. (**a**) Cortical neurons were exposed to DMSO or 20 nM TBT for various times, and then the mRNA expression of cyt.c, COX4, and COX6c was measured by real-time PCR. The data are expressed as the mean + S.E.M. (*n* = 3). * *p* < 0.05 vs. control; (**b**) Cortical neurons were exposed to 20 nM TBT for various times, and the intracellular ATP content was measured. The data are expressed as the mean + S.E.M. (*n* = 24–32). ** *p* < 0.01 vs. control.

NRF-1 is a transcription factor that increases the expression of not only *GluR2*, but also genes related to mitochondrial function, such as *cytochrome c (cyt.c)*, *cytochrome c oxidase (COX)*, and *mitochondrial transcription factor A (TFAM)* [27,28,29]. To determine whether TBT decreases the mRNA expression of other genes downstream from NRF-1 by inhibiting NRF-1 activity, rat primary cortical neurons were exposed to 20 nM TBT for 3 h, 24 h, and 9 days, and the mRNA expression of COX4, COX6c, and cyt.c was evaluated. As shown in Figure 3a, the mRNA expression of COX4, COX6c, and cyt.c was decreased to 51%, 46%, and 55%, respectively, relative to the control, following exposure to 20 nM TBT for 9 days. Next, we measured the adenosine triphosphate (ATP) content in the cortical neurons, because COX4, COX6c, and cyt.c are related to ATP production through oxidative phosphorylation in the mitochondria. Consistent with the decreased mRNA expression of these three genes, the ATP content was decreased to 88% relative to the control following exposure to 20 nM TBT for 9 days (Figure 3b). These results suggest that TBT decreases the mRNA expression of COX4, COX6c, and cyt.c by inhibiting NRF-1, which may result in a moderate but significant decrease in intracellular ATP content.

### 2.4. Effect of TBT on Expression of NRF-1 and PGC-1α

**Figure 4:**
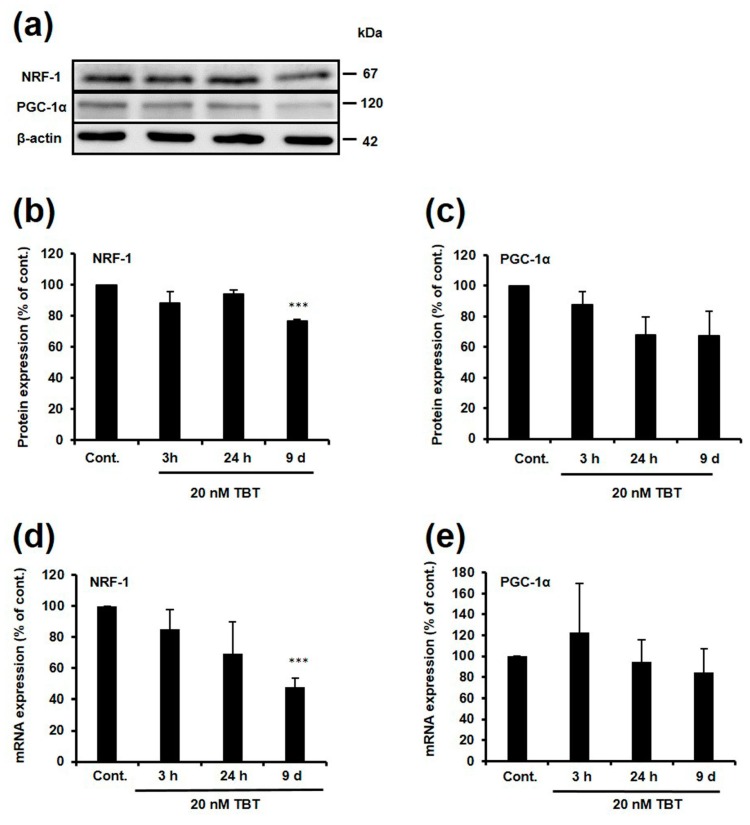
Effect of TBT on NRF-1 and PGC-1α expression. (**a**) Cortical neurons were exposed to DMSO or 20 nM TBT for various times, then NRF-1 and PGC-1α protein expression was detected by western blotting. Quantitative analysis of the NRF-1 (**b**) and PGC-1α (**c**) protein expression was performed using Image J software, and protein levels were corrected based on β-actin protein levels. The data are expressed as the mean + S.E.M. (*n* = 3). *** *p* < 0.001 vs. control. Cortical neurons were exposed to DMSO or 20 nM TBT for various times, and then NRF-1 (**d**) and PGC-1α (**e**) mRNA expression was measured by real-time PCR. The data are expressed as the mean + S.E.M. (*n* = 4). *** *p* < 0.001 vs. control.

Peroxisome proliferator-activated receptor gamma coactivator 1-alpha (PGC-1α) is a transcriptional coactivator that strictly controls gene expression by binding to specific transcription factors, including NRF-1 [30,31]. If TBT-induced NRF-1 inhibition is caused by a decrease in either NRF-1 or PGC-1α protein expression, a decrease in the expression of these proteins would be observed at least 3 h after TBT exposure. To investigate whether the expression of NRF-1 and PGC-1α was changed following TBT exposure, we performed a western blot and real time PCR using rat primary cortical neurons. Exposure of the cells to 20 nM TBT for 9 days resulted in a significant decrease of NRF-1 protein and mRNA expression to 77% and 48% relative to the control, respectively (Figure 4a,b,d). In addition, PGC-1α protein and mRNA expression was modestly and non-significantly decreased to 67% and 85% following exposure to 20 nM TBT for 9 days, respectively (Figure 4a,c,e). Although NRF-1 and PGC-1α expression was decreased by TBT exposure for 9 days, this is not the main mechanism of NRF-1 inhibition by TBT.

### 2.5. Effect of TBT on Nuclear and Cytosolic Localization of NRF-1 and PGC-1α, and Dimerization of NRF-1

**Figure 5:**
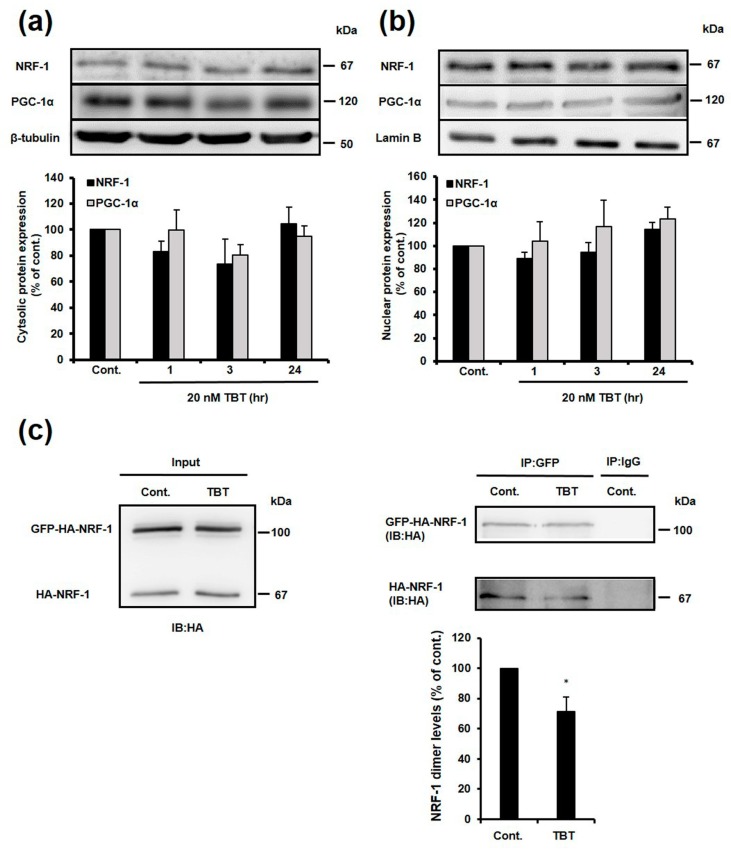
Effect of TBT on NRF-1 and PGC-1α nuclear translocation. Cytosolic proteins (**a**) and nuclear proteins (**b**) were extracted from DMSO- or TBT-treated cortical neurons, and then NRF-1 and PGC-1α protein expression was detected by western blotting. Quantitative analysis was performed using Image J software, and the protein levels were corrected based on the β-tubulin and Lamin B protein levels. The data are expressed as the mean + S.E.M (*n* = 3); (**c**) HEK 293T cells, co-expressing GFP-HA-tagged NRF-1 (GFP-HA-NRF-1) and HA-tagged NRF-1 (HA-NRF-1), were exposed to 20 nM TBT for 3 h, and total cell extracts were prepared. Crude proteins were immunoprecipitated with anti-GFP antibody and normal mouse IgG antibody (negative control), and western blotting was performed using anti-HA antibody. The immunoprecipitated HA-NRF-1 levels were corrected relative to the immunoprecipitated GFP-HA-NRF-1 levels. The data are expressed as mean + S.E.M. (*n* = 3). * *p* < 0.05 vs. control.

To identify the mechanisms of NRF-1 inhibition, we examined whether the nuclear localization of NRF-1 and PGC-1α was affected by TBT exposure, because nuclear localization is important for regulation of downstream gene expression. Rat primary cortical neurons were exposed to 20 nM TBT for 1, 3 and 24 h, and NRF-1 and PGC-1α protein expression was evaluated in the nuclei and cytoplasm. As shown in Figure 5a,b, nuclear and cytosolic localization of NRF-1 and PGC-1α was not significantly changed by TBT exposure. These results suggest that nuclear localization of NRF-1 and PGC-1α was not affected by TBT exposure.

Next, we examined whether TBT inhibits the homo-dimerization of NRF-1 by using human embryonic kidney (HEK) 293T expression system, because the homo-dimerization of NRF-1 is crucial for its ability to bind DNA [32]. As shown in Figure 5c, the NRF-1 dimer levels were decreased by exposure to 20 nM TBT for 3 h. This result raises the possibility that a decrease in NRF-1 transcriptional activity in primary neurons can result from the inhibition of NRF-1 homo-dimerization by TBT.

## 3. Discussion

In this study, cultured rat cortical neurons were exposed to a low level of TBT (20 nM) to identify the mechanism via which TBT induces a decrease in *GluR2* expression. The expression of the *GluR2* protein was decreased in both the whole cell and the plasma membrane following exposure to 20 nM TBT for 9 days (Figure 1). This will subsequently enhance Ca^2+^ permeability and increase neuronal vulnerability as shown in Figure S1 and previous report [16]. *GluR2* mRNA expression was also decreased by TBT exposure, indicating that the TBT-induced decrease in *GluR2* protein expression is a result of transcriptional inhibition of the *GluR2* gene. There is a time lag between when the decrease in protein expression is observed (3 days), and when the decrease in mRNA expression is observed (3 h). This time lag can be explained by the long half-life of the *GluR2* protein, which has been reported to be 5–7 days [33].

It is known that *GluR2* expression is regulated by a variety of transcription factors, including NRF-1, Sp1, and REST [26]. In addition to its role in regulating *GluR2* expression, NRF-1 also binds to the *cyt.c* promoter [34], and regulates the expression of genes involved in mitochondrial function and cell proliferation, such as *COX* and *TFAM* [27,28,29]. Furthermore, NRF-1 specifically binds to and activates the *GluR2* promoter, without activating the promoters of the other AMPA receptor subunits, *GluR1*, *GluR3*, and *GluR4* [35]. Sp1 is a general transcription factor that binds to and acts through GC-boxes. It is involved in the expression of many different genes [36], and has been reported to bind and activate the promoters of all of the AMPA receptor subunits [37,38]. REST is a repressor that negatively regulates the expression of many neuron-specific genes, including *GluR2* [26,39], and it is assumed that REST is involved in neuronal cell death through the AMPA receptor [40]. In the present study, we showed that TBT specifically inhibits the DNA binding activity of NRF-1, without affecting the other *GluR2* transcription factors, Sp1 and REST (Figure 2).

**Figure 6:**
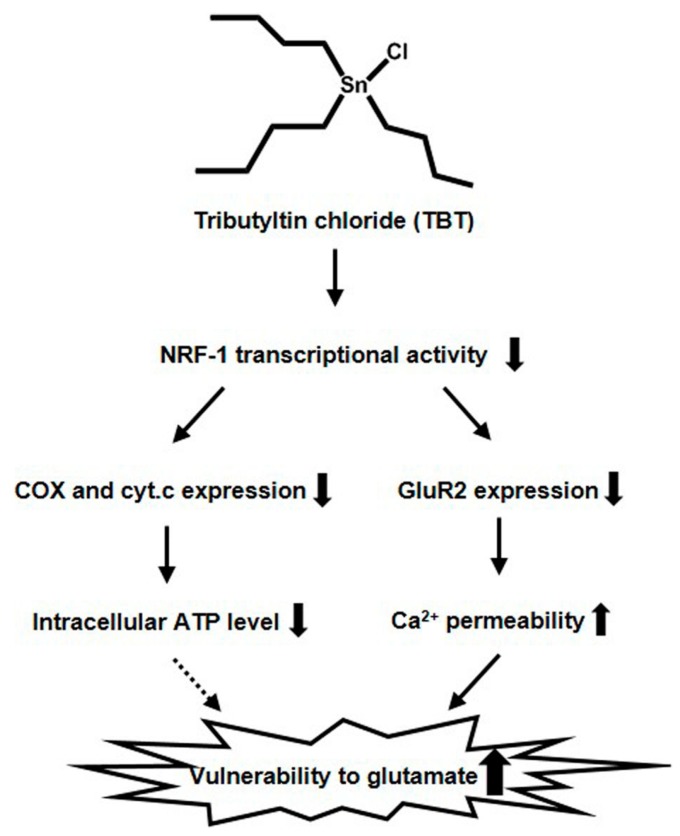
Schematic representation of the proposed mechanism by which TBT induces neurotoxicity via NRF-1 inhibition in primary cortical neurons. 20 nM TBT inhibits the transcriptional activity of NRF-1, and subsequently decreases the expression of downstream genes, including *GluR2* and mitochondrial related genes (cyt.c, COX4, and COX6c). The decrease in expression of *GluR2* and the mitochondrial related genes results in an increase in neuronal Ca^2+^ permeability and a decrease in the intracellular ATP level, respectively, which ultimately enhances neuronal vulnerability to glutamate excitotoxicity. Solid arrows indicate the identified pathways. Dashed arrow indicates the hypothetical pathway. Solid lines thick arrows (up) indicate the increase. Solid lines thick arrows (down) indicate the decrease.

NRF-1 positively regulates the expression of cyt.c, COX, and NMDA receptor subunit 1 (NR1), in addition to *GluR2* [41]. In this work, we showed that exposure of primary rat cortical neurons to 20 nM TBT resulted in decreased mRNA expression of cyt.c, COX4, and COX6c (Figure 3a), and TBT has been shown to decrease the mRNA expression of NR1 in a previous report [16]. This provides further evidence that the transcriptional activity of NRF-1 is inhibited by TBT exposure. Moreover, we evaluated the ATP content in TBT-exposed neurons, because cyt.c and COX are involved in the process of ATP production through mitochondrial oxidative phosphorylation. As shown in Figure 3b, a slight but significant decrease in ATP content was observed following TBT exposure. Although it has been previously reported that TBT decreases ATP content by inhibiting mitochondrial function, the concentration of TBT used in these previous studies was high and resulted in cell death [42,43]. Our results suggest that low-concentration TBT can induce modest ATP reduction, at least partly by depressing mitochondrial activity due to NRF-1 inhibition, without causing neuronal cell death. (Figure 6).

PGC-1α directly or indirectly regulates NRF-1 transcriptional activity, and affects downstream gene expression [30,31]. If TBT inhibits the transcriptional activity of NRF-1 by decreasing NRF-1 or PGC-1α protein expression, a decrease in the expression of these genes would be observed at least 3 h after TBT exposure. Therefore, we investigated the effect of TBT on PGC-1α expression. Although the protein and mRNA expression of NRF-1 were significantly decreased (Figure 4a,b,d), and the expression of PGC-1α was also decreased (Figure 4a,c,e) following exposure to 20 nM TBT for 9 days, a decrease in NRF-1 protein expression is not considered to be the main mechanism of TBT-induced inhibition of NRF-1 transcription activity, because there is a time lag between when inhibition of NRF-1 transcription activity was observed in the ChIP assay (Figure 2e), and when a decrease in protein expression was observed. We examined the nuclear expression of NRF-1 and PGC-1α to further identify the mechanism of TBT-induced NRF-1 inhibition. Unexpectedly, the nuclear expression of NRF-1 and PGC-1α was not decreased but slightly increased in a time dependent manner following TBT exposure (Figure 5), possibly because of an unknown protective effect that was activated in response to the decrease in NRF-1 transcription activity.

It is reported that a homodimer is the active binding species of NRF-1 [32]. Here, we showed that TBT inhibited dimerization of NRF-1 by using HEK 293T expression system (Figure 5c). Although we should investigate the effect of TBT on endogenous NRF-1 formation in primary neurons to confirm this finding, a decrease in NRF-1 homo-dimerization can be involved in the inhibition of NRF-1 transcriptional activity by TBT. The mechanism of NRF-1 activation has been reported in some studies. Izumi et al. reported that sodium butyrate increased the acetylation of NRF-1 via p300/CBP associated factor, and activated the promoter of the *N*-acetylgalactosaminyltransferase-3 gene without changing the nuclear content of NRF-1 [44]. Moreover, serine phosphorylation within the *N*-terminal of NRF-1 is also important for the DNA binding activity of NRF-1 [32]. Cyclin D1-dependent kinase phosphorylates S47 of NRF-1, which inhibits NRF-1 dependent functions [45]. TBT may inhibit NRF-1 by affecting kinases like cyclin D1-dependent kinase, and subsequently affecting the serine phosphorylation of NRF-1. To clarify the mechanisms of TBT-induced NRF-1 inhibition, post-transcriptional modification of NRF-1 should be examined following TBT exposure in further studies.

Overexpression of NRF-1 increases neurite elongation in human neuroblastoma cells and mouse primary cortical neurons, but a dominant-negative mutant of NRF-1 decreases neurite elongation [46]. NRF-1 knockout (KO) mice die between embryonic days 3.5 and 6.5. This coincides with a decrease in mitochondrial DNA (mtDNA) expression, which is regulated by TFAM [47]. Although it is plausible that embryonic death induced by NRF-1 disruption is due to a decrease in mtDNA levels caused by a decrease in TFAM, TFAM KO mice die after embryonic day 8.5 [48]. Therefore, Huo and Scarpulla proposed that embryonic death induced by NRF-1 disruption is affected by not only TFAM, but also other known or unknown NRF-1 target genes. Therefore, the decrease in NRF-1 transcriptional activity induced by a low level of TBT may cause neurotoxicity through a decrease in not only *GluR2*, but also other NRF-1 target genes.

## 4. Materials and Methods

### 4.1. Materials

Eagle’s minimal essential salt medium (Eagle’s MEM) was purchased from Nissui Pharmaceutical (Tokyo, Japan). Penicillin G and streptomycin sulfate were purchased from Meiji Seika (Tokyo, Japan). Fetal calf serum (FCS) was purchased from Nichirei Biosciences Inc. (Tokyo, Japan). Horse serum (HS) was purchased from Gibco (Life Technologies, Carlsbad, CA, USA). d-(+)-glucose, NaHCO_3_, sodium orthovanadate, sodium dodecyl sulfate (SDS), glycerol, sodium deoxycholate, and tributyltin chloride were purchased from Wako (Tokyo, Japan). l-Glutamine, arabinosylcytosine, formaldehyde, Triton X-100, MgCl_2_, and anti-β-actin antibody (AC-15) were purchased from Sigma-Aldrich (St. Louis, MO, USA). NaHCO_3_ was purchased from Kanto Chemical (Tokyo, Japan). Pentobarbital was purchased from Kyoritsu (Tokyo, Japan). Bromophenol blue was purchased from Katayama Chemical Industries Co., Ltd. (Osaka, Japan). Tris-HCl, nonidet P-40, ethylenediaminetetraacetic acid (EDTA), 4-(2-hydroxyethyl)-1-piperazineethanesulfonic acid (HEPES), mercaptoethanol, dithiothreitol (DTT), KCl, and protease inhibitor cocktail were purchased from Nacalai Tesque (Kyoto, Japan). Anti-NRF-1 antibody chip-grade (ab34682) was purchased from Abcam (Cambridge, UK). Anti-NRF-1 antibody (#H00004899-M01) was purchased from Abnova (Taipei, Taiwan). Anti-*GluR2* antibody (MAB397) was purchased from Millipore (Billerica, MA, USA). Anti-*N*-cadherin antibody (sc-7939), anti-PGC-1α antibody (sc-13067), and anti-lamin B antibody (sc-6217) were purchased from Santa Cruz Biotechnology (Dallas, TX, USA).

### 4.2. Cortical Neuron Culture

The study was approved by the animal ethics committee at Hiroshima University. The following procedures were performed under sterile conditions. The prefrontal part of the cerebral cortex was dissected from fetal rats at gestation day 18, and the cells were dissociated by gentle pipetting and plated onto culture plates.

(4 × 10^5^ cells/cm^2^). The cultures were incubated in Eagle’s MEM supplemented with 10% FCS, l-glutamine (2 mM), d-(+)-glucose (11 mM), NaHCO_3_ (24 mM), and HEPES (10 mM). The cultures were maintained at 37 °C with 5% CO_2_ in MEM containing 10% FCS (days in vitro (DIV) 1–7) or 10% HS (DIV 8–11), and the medium was changed every 2 days. Arabinosylcytosine (10 μM) was added after DIV 6, and the cultures were used for experiments at DIV 11. This protocol has been confirmed to produce cultures containing approximately 90% neurons (Figure S2).

### 4.3. Protein Isolation

#### 4.3.1. Whole-Cell Extracts

After TBT treatment, the cells were washed with PBS buffer and lysed in Tris-ethylenediaminetetraacetic acid sodium chloride (TNE buffer) containing 50 mM Tris-HCl, 1% nonidet P-40, 20 mM EDTA, protease inhibitor cocktail (1:100), 1 mM sodium orthovanadate, and 1 mM PMSF. The mixture was rotated at 4 °C and centrifuged at 15,000 rpm, after which the supernatant was transferred to a microtube.

#### 4.3.2. Nuclear Extracts

After TBT exposure, the cells were washed with PBS buffer and collected in a microtube. After centrifugation, the cell pellet was resuspended in hypotonic buffer (10 mM HEPES, 1.5 mM MgCl_2_, 10 mM KCl, 1 mM DTT, protease inhibitor cocktail [1:100]). After being left for 15 min on ice, 0.5% NP-40 was added, and the cell suspension was vortexed and centrifuged at 10,000× *g* for 10 min. The supernatant was then transferred to a microtube (cytoplasmic extract). The cell pellet was subsequently resuspended in hypertonic buffer (20 mM HEPES, 1.5 mM MgCl_2_, 400 mM NaCl, 0.1 mM EDTA, 10% glycerol, 1 mM DTT, protease inhibitor cocktail [1:100]). After sonication, the mixture was centrifuged at 12,000× *g* for 10 min. The supernatant was then transferred to a microtube (nuclear extract).

#### 4.3.3. Membrane Extract

After the cells were washed with PBS buffer, 0.5 mg/mL (PBS (+)) EZ-Link^®^ Sulfo-NHS-Biotin solution (Thermo, San Jose, CA, USA) was added to the culture dish, and the cells were incubated for 20 min at 4 °C. Then, to stop the biotinylation reaction, PBS containing 100 mM glycine was added to the culture dish, and the cells were incubated for 30 min at 4 °C. The cells were then lysed in TNE buffer, and transferred to a microtube. The mixture was rotated at 4 °C and centrifuged at 15,000 rpm for 15 min. The supernatant was incubated with PBS containing avidin-agarose beads (SIGMA, St. Louis, MO, USA) at 4 °C overnight. After centrifugation at 15,000 rpm for 15 min, the pellet was resuspended in sample buffer, and denatured at 70 °C for 30 min to cleave the membrane protein–biotin bonds. The sample was then analyzed by western blotting.

### 4.4. Western Blotting

Western blotting was performed as previously described [49] with some modifications. Denaturing buffer (100 mM Tris-HCl, 4% SDS, 20% glycerol, 0.004% bromophenol blue, and 5% mercaptoethanol) was added to the protein extracts, and the extracts were incubated at 95 °C for 3 min. The extracts were then separated by electrophoresis, and transferred to a polyvinylidene difluoride membrane. The membrane was blocked with 5% skim milk for 1 h, and then incubated with the primary antibody overnight, before being incubated with the secondary antibody for 1 h. The proteins were detected using an enhanced chemiluminescent detection system (Chemi-Lumi One L; Nacalai Tesque, Kyoto, Japan), and quantified using digital imaging software (Image J; NIH, Bethesda, MD, USA).

### 4.5. Quantitative Real-Time PCR

Total RNA was extracted using an SV Total RNA Isolation System (Promega, Madison, WI, USA) according to the manufacture’s protocol. The total RNA was then reverse-transcribed at 42 °C for 1 h, and 75 °C for 15 min, in a reaction mixture containing 5× reaction buffer, RNase inhibitor, reverse transcriptase, and dNTP. Quantitative RT-PCR was performed using a QuantiTect SYBR Green PCR Kit (Qiagen, Valencia, CA, USA). The thermocycling conditions were as follows: 95 °C for 3 min, then 50 cycles of 95 °C for 15 s, 60 °C for 30 s, and 72 °C for 30 s. The mRNA levels were normalized to glyceraldehyde-3-phosphate dehydrogenase (GAPDH). The primers used for the PCR are shown in Table S1.

### 4.6. Immunocytochemistry

Detailed protocols for immunocytochemistry are included in Supplementary Information.

### 4.7. ChIP Assay

Detailed protocols for ChIP assay are included in Supplementary Information.

### 4.8. Electrophoresis Mobility Shift Assay (EMSA)

Oligonucleotide probes containing the NRF-1 binding site on the *GluR2* promoter (Table S1) were annealed, and with [γ^32^P] ATP (3000 Ci/mmol at 10 mCi/mL; Perkin-Elmer, Shelton, CT, USA). The oligonucleotides were then separated from the unincorporated nucleotides by chromatography using a Micro Spin G-25 column (GE Healthcare, Waukesha, WI, USA). The purified probes were incubated with 5 μg of nuclear extract, 5× binding buffer (Promega), and gel loading buffer. For competition, oligonucleotides were incubated with the nuclear extract before adding the oligonucleotides. The probe/nuclear extract mixture was loaded onto a 4% polyacrylamide gel, and run at 200 V for 60–90 min. The results were visualized by autoradiography.

### 4.9. Plasmid Constructions

Full-length HA-tagged NRF-1 was generated using the primers summarized in table S1. The PCR product was digested with Hind III and BamHI and cloned into the pcDNA3.1/Zeo (+) (Invitrogen, Carlsbad, CA, USA) vector and pAcGFP-C1 vector (Takara bio Inc., Otsu, Japan) cut with these two enzymes. All clones generated by PCR were confirmed by sequencing.

### 4.10. Human Embryonic Kidney (HEK) 293T Cell Culture and Transfection

HEK 293T cells were cultured in DMEM (Nissui) supplemented with 10% FBS, 0.58 mg/mL l-glutamine, 2 mg/mL NaHCO_3_, 100 units/mL penicillin (Meiji Seika Pharma, Tokyo, Japan), 100 μg/mL streptomycin (Meiji Seika Pharma, Tokyo, Japan), and 4.5 g/L at 37 °C in a humidified 5% CO_2_ incubator. For transfection of pcDNA3.1 and pAcGFP-C1 plasmids, FuGENE HD (Promega) was used as recommended by the manufacturer. After transfection for 48 h, cells were exposed to 20 nM TBT and lysed in TNE buffer.

### 4.11. Immunoprecipitation

Detailed protocols for immunoprecipitation are included in Supplementary Information.

### 4.12. ATP Measurement

After exposure to TBT, the intracellular ATP content was measured using a Cell Titer-Glo^®^ Luminescent Cell Viability Assay (Promega), according to the manufacture’s protocol.

### 4.13. Statistics Analysis

All the experiments were performed at least three times and representative data are shown. Data are expressed as mean + S.E.M. Statistical evaluation of the data was performed with ANOVA followed by Turkey’s test or with Student’s *t*-test. A value of *p* < 0.05 was considered to be indicative of significance.

## Supplementary Materials

Supplementary materials can be found at www.mdpi.com/1422-0067/18/8/1754/s1.
