# Supplementary material for: Low-Concentration Tributyltin Decreases GluR2 Expression via Nuclear Respiratory Factor-1 Inhibition
Source: Int J Mol Sci. 2017 Aug 11;18(8):1754. doi: 10.3390/ijms18081754 (PMC5578144; doi:10.3390/ijms18081754)
Supplement: Supplementary file 1 [file ijms-18-01754-s001.pdf]

Supplementary Information

Low-concentration tributyltin decreases GluR2 expression via nuclear respiratory factor-1 inhibition

Keishi Ishida, Kaori Aoki, Tomoko Takishita, Masatsugu Miyama, Shuichiro Sakamoto, Seigo Sanoh, Tomoki Kimura, Yasunari Kanda, Shigeru Ohta, and Yaichiro Kotake

Materials and Methods

Immunocytochemistry

Cells were seeded onto polyethyleneimine-coated glass coverslips, and incubated overnight. After treatment with 20 nM TBT for 9 days, the cells were washed with PBS(-), and fixed with 4% paraformaldehyde in PBS(-) for 10 min at room temperature. The glass coverslips were then washed with PBS(-), blocked with 2 drops of Image-iT® FX Signal Enhancer (Molecular Probes®) for 1 h, and incubated with mouse anti-GluR2, which recognizes the N-terminal extracellular domain of GluR2 (1:250), rabbit anti-N-cadherin (1:250), mouse anti-GFAP (1:200, Sigma-Aldrich), and rabbit anti-Tuj1 (1:400, Covance Research Products) diluted in PBS(-) for 1 h at room temperature. Then, the glass coverslips were washed three times with PBS(-), and incubated with Alexa Fluor® 488-conjugated goat anti-mouse IgG (1:500, Molecular Probes®) and Alexa Fluor® 555-conjugated goat anti-rabbit IgG (1:500, Molecular Probes®) for 1 h at room temperature in the dark. The glass coverslips were further washed three times with PBS(-). Finally, the glass coverslips were enclosed in Prolong® Diamond reagent (Molecular Probes®), and observed under a confocal laser scanning microscope (Carl Zeiss, LSM5 PASCAL).

ChIP assay

TBT-treated cells were incubated with 1% formaldehyde to crosslink the protein–DNA complexes. The fixation was then quenched by incubating the cells with 0.125 M glycine. The cells were then washed with ice-cold PBS, and suspended with 100 μL cell lysis buffer (10 mM HEPES, 1.5 mM $MgCl_2$, 10 mM KCl, 0.5 mM DTT, 0.2 mM PMSF). After being left on ice for 10 min, the mixture was homogenized with 15 strokes. The homogenate was then transferred to another microtube, and centrifuged at 6,300 rpm for 10 min. The nuclear pellet was resuspended in 150 μL sonication buffer (50 mM Tris, 10 mM EDTA, 1% SDS, protease inhibitor cocktail), and sonicated on ice, resulting in the formation of genomic DNA fragments (200–1000 bp). The nuclear lysates were centrifuged at 12,000 rpm for 10 min to remove any insoluble material. The resulting lysates were then immunoprecipitated overnight at 4 °C using Protein G Dynabead (Invitrogen)/NRF-1 antibody (Abcam) complexes. The Dynabead–antibody–antigen complexes were sequentially washed with ChIP washing buffer 1 (16.7 mM Tris, 0.01% SDS, 1% Triton X-100, 1.2 mM EDTA, 150 mM NaCl), ChIP washing buffer 2 (twice) (16.7 mM Tris, 0.01% SDS, 1% Triton X-100, 1.2 mM EDTA, 500 mM NaCl), LiCl washing buffer (10 mM Tris, 250 mM LiCl, 1% NP40, 1% sodium deoxycholate, 1 mM EDTA), and TE buffer (twice) (10 mM Tris, 1 mM EDTA). The Dynabead–antibody–antigen complexes were then suspended in 250 μL of elution buffer (1% SDS, 50 mM

$NaHCO_3$, 10 ng/mL sheared salmon sperm DNA (Thermo)), and incubated for 15 min at room temperature with rotation. After this, the supernatant was transferred to a microtube. The Dynabead–antibody–antigen complexes were suspended again in 250 μL elution buffer, and transferred to another microtube. The ChIP samples and input samples (reserved before immunoprecipitation) were incubated in high salt conditions (added to 200 mM NaCl) at 65 °C overnight for crosslink reversal. The DNA fragments were then purified by suspending in 500 μL phenol/chloroform/isoamylalcohol, and the upper layer was transferred to a new microtube. The purified DNA samples were then subjected to quantitative real-time PCR analyses (OPTICON3). The ChIP data were analyzed using the ΔΔCt method, and normalized to the input samples. The PCR primers are shown in Supplementary Table S1.

Measurement of cell viability.

After exposure to TBT or glutamate, neurons were stained with 0.4% trypan blue for 10 min, then fixed with 10% formalin for 2 min and rinsed with physiological saline. Stained cells were regarded as dead and unstained cells were regarded as viable. Cell viability was then calculated as the percentage ratio of unstained cells to total cells counted. Over 200 cells per well were randomly counted.

Immunoprecipitation

Protein G dynabeads (Invitrogen) were transferred to microtube and washed twice with PBS-T (PBS with 0.1% Tween 20), after which the supernatant was removed. 200 μL PBS-T containing 1 μg of anti-green fluorescent protein (GFP) antibody (Santa Cruz, sc-9996) or anti-normal mouse IgG antibody (Millipore, 12-371) was added to microtube and incubated with rotation for 10 min. After the dynabeads-antibody (Ab) complex was washed with PBS-T, crude protein (200 μg) was rotated with the dynabeads-Ab complex for 1 h. Dynabeads-Ab-antigen (Ag) complex was washed 3 times with PBS-T and transferred to another microtube to avoid co-elution of proteins that bound to the tube wall. After removed supernatant, 20 μL of a sample buffer was added to the microtube and then denatured at 95°C for 3 min to elute proteins. To analyze the sample, western blotting was performed using an anti-HA antibody (MBL, 561).

**Experiment**	**Gene**	**Sequence (5' to 3')**
real-time PCR	GluR2	F: aacgagtacatcgagcagaggaa
		R: gatgccgtagcctttggaatc
	COX4	F: cacttcggtgtgccttcggg
		R: aaaggctgctccagtcggc
	COX6c	F: agcgtctgcgggttcata
		R: gcctgcctcatctcttcaaa
	cyt.c	F: ggcaagcataagactggaccaa
		R: tttccaaatactccatcagggtatc
	NRF-1	F: aaaaggcctcatgtgtttgagt
		R: agggtgagatgcagagaacaat
	PGC-1α	F: caatgaatgcagcggtctta
		R: acgtctttgtggcttttgct
	GAPDH	F: ctcgtctcatagacaagatggtgaag
		R: agactccacgacatactcagcacc
ChIP assay	GluR2	F: cgcaagactggaggtctctaa
		R: cgcacacacacaggaaagtc
EMSA	NRF-1	F: ttttgcctcgagtcgcgcacgcgcgcccgggactgc
		R: ttttgcagtcccgggcgcgcgtgcgcgactcgaggc
	REST	F: agctccgactaaagcgctgtcctcggtgctaaaatcgg<br>R: ccgattttagcaccgaggacagcgctttagtcggagct
	Sp1	F: attcgatcggggcggggcgagc<br>R: gctcgccccgccccgatcgaat
Plasmid construction	HA-NRF-1	1st F: tacccatacgatgttccagattacgctatggaggaacacggagtg
		2nd F: gtacgagacgaagcttgcaccatgtacccatacgatgttccaga
		R: gacatgattaggatcctcactgttccaatgtcaccacc

Table S1. Sequences of primers used for experiments.

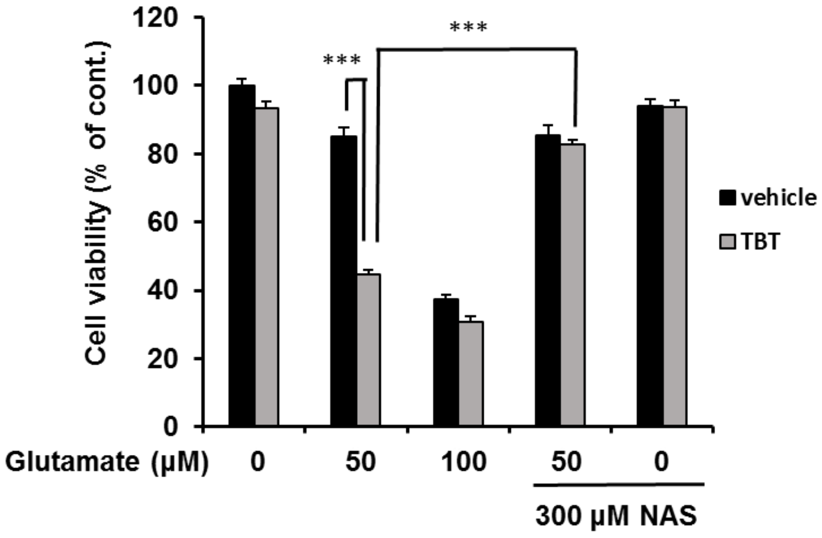

Figure S1. Effect of TBT exposure on glutamate toxicity in primary cortical neurons.

Cortical neurons were exposed to 50-100 μM glutamate for 24h with or without 300 μM 1-naphtyl acetyl spermine (NAS) after TBT exposure and cell viability was measured. The data are expressed as the mean + S.E.M. (*n*=4). ***P < 0.001 vs. control.

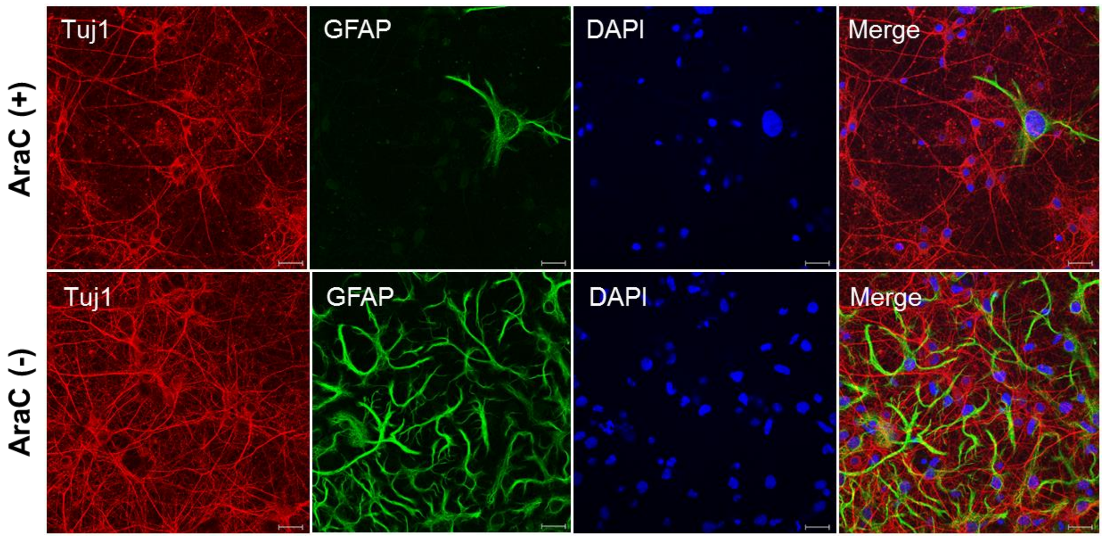

Figure S2. Immunocytochemistry of neuronal and glial cells of the primary culture on DIV 11. To evaluate neuronal and glial populations, cultures were grown to DIV 11 with or without arabinosylcytosine (AraC) to inhibit the proliferation of glial cells. Then, immunostaining was performed using an anti-Tuj1 (neuronal marker) antibody (red) and an anti-GFAP (glial marker) antibody (green). 4',6-Diamidino-2-phenylindole (DAPI) was used for nuclear staining (blue); scale bar = 20 μm.
